# Supplementary material for: Sub-Optimal Paternal Diet at the Time of Mating Disrupts Maternal Adaptations to Pregnancy in the Late Gestation Mouse
Source: Nutrients. 2024 Jun 14;16(12):1879. doi: 10.3390/nu16121879 (PMC11206308; doi:10.3390/nu16121879)
Supplement: Supplementary file 1 [file nutrients-16-01879-s001.zip › Supplementary table S1.pdf]

Supplementary Table S1: Ingredients and nutritional information of diets fed to male mice

	**CD**	**LPD**	**MD-LPD**	**WD** †	**MD-WD**
**Energy density (kcal/g)**	3.10	3.10	3.10	4.63	4.63
**Proportional energy content (%)**					
Protein	16	8.4	8.4	14.7	14.7
Fat	23.4	23.1	23.1	41.4	41.4
Carbohydrate	60.5	68.5	68.5	43.9	43.9
of which sugars	20.7	23.6	23.6	34.6	34.6
**Protein (% g/g)**					
Casein	18.0	9.0	9.0	19.5	19.5
**Fats (% g/g)**					
Corn Oil	10	10	10	1	1
Milk fat	-	-	-	20	20
Cholesterol	-	-	-	0.15	0.15
**Carbohydrates (% g/g)**					
Sucrose	21.3	24.3	24.3	33.9	33.9
Starch Maize	42.5	48.5	46.0	15.0	12.3
Cellulose	5	5	5	5	5
**Micronutrients (% g/g)**					
Choline chloride	0.2	0.2	0.7	0.2	0.7
D,L-Methionine	0.50	0.50	1.25	0.00	0.75
Betaine	-	-	1.5	-	1.5
Mineral mix (AIN-76)	2	2	2	3.5	3.5
Vitamin mix (AIN-76)	0.5	0.5	0.5	1.0	1.0
* Folic Acid	1×10^{-4}	1×10^{-4}	1.6×10^{-3}	2×10^{-4}	1.6×10^{-6}
* Vitamin B12	5×10^{-7}	5×10^{-7}	1.5×10^{-4}	1×10^{-6}	1.5×10^{-4}

* Components contained within commercially available Vitamin mix AIN-76 that were supplemented in methyl-donor diets.

† WD is commercially manufactured by Special Diet Services, diet code 829100. All other diets are custom formulations from Special Diet Services.
