# Supplementary material for: Sub-Optimal Paternal Diet at the Time of Mating Disrupts Maternal Adaptations to Pregnancy in the Late Gestation Mouse
Source: Nutrients. 2024 Jun 14;16(12):1879. doi: 10.3390/nu16121879 (PMC11206308; doi:10.3390/nu16121879)
Supplement: Supplementary file 1 [file nutrients-16-01879-s001.zip › Supplementary table S2.pdf]

Supplementary Table S2: Information on primer sequences for gene expression RT-qPCR

Gene Name	Gene Symbol	Accession Number	Forward Primer	Reverse Primer	Amplicon Length
Phosphoglycerate kinase 1	*Pgk1*	NM_008828	TACCTGCTGGCTGGATGG	CACAGCCTCGGCATATTTCT	65 bp
Tubulin a	*Tuba*	NM_011653	CTGGAACCCACGGTCATC	GTGGCCACGAGCATAGTTATT	114 bp
Angiotensin I Converting Enzyme 2	*Ace2*	NM_207624.5	TGTAGAACGTACCTTCGCAGAG	GGGCTGATGTAGGAAGGGTA	99 bp
Adrenoceptor Beta 1	*Adrb1*	NM_007419.2	GGATCGCCTCTTCGTCTTCT	CAGTAGATGATGGGGTTGAAGG	69 bp
Angiotensin II receptor, type 1a	*Agtr1a*	NM_177322.3	ACTCACAGCAACCCTCCAAG	CTCAGACACTGTTCAAAATGCAC	62 bp
ATPase Plasma Membrane Ca2+ Transporting 1	*Atp2b1*	NM_026482.2	AACCTCCGGAAGGGGATAAT	AATCCACCCCGTTTCTCCT	80 bp
